# Lupin Seed Supplementation as a Functional Feed Additive: *In Vitro* Ruminal Gas, Methane and Carbon Dioxide Production, Fermentation Kinetics, and Nutrient Degradability

**DOI:** 10.3390/ani14142119

**Published:** 2024-07-20

**Authors:** Tarek A. Morsy, Ahmed E. Kholif, Moyòsore J. Adegbeye, Olurotimi A. Olafadehan, Gouda A. Gouda, Mahmoud Fahmy, Mireille Chahine

**Affiliations:** 1 Dairy Science Department, National Research Centre, 33 Bohouth St. Dokki, Giza 12622, Egypt; tarekalymo@gmail.com (T.A.M.); gagouda@gmail.com (G.A.G.); fahmymahmoud2@gmail.com (M.F.); 2 Department of Animal Sciences, North Carolina Agricultural and Technical State University, Greensboro, NC 27411, USA; 3 Department of Animal Production and Health, University of Africa, Toru-Orua, Sagbama 561101, Nigeria; alanspeco@yahoo.com; 4 Department of Animal Science, University of Abuja, Abuja 900105, Nigeria; oaolafadehan@yahoo.com; 5 Department of Animal, Veterinary and Food Sciences, University of Idaho, 315 Falls Ave., Twin Falls, ID 83301, USA

**Keywords:** degradability, functional feed additives, *in vitro* fermentation, lupin seed, methane, phytogenics

## Abstract

**Simple Summary:**

Enteric greenhouse gas (GHG) emission remains a challenge in modern livestock production as it relates environmental perspectives to animal production. In view of this, constant attempts are made to use ingredients that reduce enteric GHG emission and improve energy efficiency. This study aimed to investigate the effect of lupin seeds as a functional feed additive on *in vitro* ruminal fermentation, and methane (CH_4_) and carbon dioxide (CO_2_) emissions. Lupin seed lowered the proportion of CH_4_ in the total biogas production. Also, lupin seed increased fiber digestibility, total short-chain fatty acids, acetic acid, propionic acid, and microbial protein. Lupin seed at 2.0% supplementation increased gas production, enhanced nutrient degradability, and reduced the proportion of methane in every volume of biogas produced. The results suggest that lupin seed can be an ecofriendly ingredient that can be added to ruminant diet.

**Abstract:**

The inevitable enteric gas emission from ruminants is considered a modern-day problem from an environmental perspective. Addressing this problem requires nutritional approaches such as the use of phytogenic additives in ruminant diets. In this regard, lupin seed (LS) can be a useful additive due to its phytochemical constituents. Therefore, this study investigated the effects of lupin (*Lupinus angustifolius*) seed supplementation as a functional and sustainable feed additive in sheep diet (50:50 concentrate-to-forage ratio) on *in vitro* gas production (GP; mL/g DM), methane (CH_4_; mL/g DM) and carbon dioxide (CO_2_; mL/g DM) emissions, fermentation parameters, and nutrient degradability (g/kg DM incubated). Gas production and CH_4_ were measured per gram of incubated dry matter (DM), degradable DM (*d*DM), degradable neutral detergent fiber (*d*NDF), and degradable acid detergent fiber (*d*ADF). Lupin seeds were included at 0 (control), 0.5, 1.0, 1.5, and 2% of the diet. The seeds contained 3.27% essential oils (DM basis), with eucalyptol as the main phytochemical. The highest GP per gram of DM and *d*DM was observed (*p* < 0.01) with 2.0% LS supplementation level. While 1.0% LS had the highest GP per gram of *d*NDF, 0.5% lupin diet had the highest GP per gram of *d*ADF. Asymptotic GP and CH_4_ emissions linearly and quadratically increased (*p* < 0.01) with increasing LS level, while lag time decreased. Despite increased CH_4_ production, the proportion of CH_4_ in total biogas was lower (*p* = 0.008) for LS treatments than the control, with the 0.5% LS showing the lowest CH_4_ proportion. Production of CO_2_ increased with lupin seed treatments, with 0.5% LS producing the highest proportion (*p* = 0.027). Degradability of DM, NDF, and ADF was greater (*p* < 0.01) for the high LS supplementation level, while 0.5% supplementation level decreased ADF degradability. Total short-chain fatty acids, acetic acid, and propionic acid increased (*p* < 0.05) with LS supplementation level, leading to a reduced acetate:propionate ratio. Rumen pH decreased (*p* = 0.036) with LS supplementation, while ammonia-N decreased (*p* = 0.045) and estimated metabolizable energy increased (*p* < 0.001) linearly. Calculated microbial protein synthesis (*p* = 0.005) and gas yield (*p* = 0.047) increased with LS supplementation level. LS supplementation at 2.0% of diet (DM basis) increased GP and CH_4_ emission (mL/g DM) and enhanced nutrient degradability, suggesting its potential use as a functional feed additive for ruminants when supplemented at a 2.0% level into diet.

## 1. Introduction

The roles of ruminant animals in food production and security cannot be overemphasized. However, ruminal fermentation has several disadvantages. It produces greenhouse gases, posing a significant environmental threat, and also causes loss of feed energy. The gas production and release are not a problem themselves but a consequence of raising ruminant animals. Therefore, the need to mitigate the harmful environmental impact of the produced and emitted greenhouse gases prompted the current study. The main produced gases are methane (CH_4_) and carbon dioxide (CO_2_). Methane generation is responsible for about 2–12% energy losses in ruminants [1], impacting productivity such as milk yield and growth. Additionally, enteric CH_4_ emissions from ruminants account for approximately 17% of the global greenhouse gas emissions [2].

The Lupinus genus has been a part of animal feed in the Mediterranean region for centuries. The newly developed “sweet” lupin varieties are characterized by low alkaloid levels (previously responsible for bitterness) and high protein content. These new varieties with robust pods and reduced alkaloids have shifted lupin use as green manure and a soiling crop to a valuable source of nutritious seeds. This shift has reignited interest in lupin as a protein source in animal diets [3]. Studies have shown that supplementing ruminants with lupins offers benefits that could improve growth and reproductive efficiency, comparable to cereal grain supplements [4].

Lupin seeds contain high levels of proteins, soluble fiber, and minerals, and a low content of starch, in addition to phytochemicals [5]. Lupin species such as *Lupinus albus*, *L. angustifolius*, and *L. luteus* have an oil content ranging from 5–10%, which is relatively low. These species are free of anti-nutritional factors like trypsin inhibitors and saponins. Lupin seed oil is notably rich in unsaturated fatty acids, including oleic and linoleic acids, which can constitute up to 80% of the oil content [6]. Furthermore, LS contains phenolic compounds, saponins, and flavonoids, whose concentrations have the ability to increase or decrease digestion [7] and mitigate CH_4_ production [8,9]. The degradability of LS protein in the rumen varies between 71 and 79%, depending on the variety. Additionally, lupin has a unique profile characterized by minimal starch, high levels of both soluble and insoluble non-starch polysaccharides (NSPs), and significant amounts of raffinose oligosaccharides. These properties can influence energy utilization and the digestion of other nutrients in a diet containing LS [4]. Lupin contains NSPs, which are primarily composed of non-cellulosic polymers and pectic polysaccharides. It also contains tocopherol, lutein, α-carotene, β-carotene, and various polyphenols [10]. In an *in vitro* experiment, Um et al. [10] showed that lupin flakes at 3, 6, and 9% of total diet enhanced rumen ammonia-N (NH_3_-N) concentrations and crude protein (CP) disappearance rate. Despite the benefits of lupin and its secondary metabolite contents, not much has been reported on its impact on greenhouse gas emissions and ruminal fermentation characteristics. However, lupin shares certain metabolites with eucalyptus plants. The main metabolite is eucalyptol, which can be used to measure the potential benefit of lupin on greenhouse gas emission. Eucalyptus leaf meal contains eucalyptol and *p*-cymene, which are active ingredients in lupins that decreased CH_4_ production by 29% [11]. Sallam et al. [12] and Kumar et al. [13] demonstrated that eucalyptus oils inhibited CH_4_ production and decreased the number of ruminal protozoa. Additionally, supplementation of LS in dairy cow diets reduced ruminal protozoal population [14], which may indirectly lower methanogens by decreasing the population and activity of protozoal-associated methanogens [15]. The active ingredients, such as monoterpenes (camphor and eucalyptol), in LS can halt the growth of some microbes, although their effect against certain rumen microbes may be limited [16]. This demonstrates the ability of lupin and its secondary metabolites to manipulate rumen microbes.

The objective of this experiment was to assess the potential use of dried lupin (*Lupinus angustifolius*) seeds as a functional feed additive for ruminants, when supplemented at increasing levels to a total mixed ration (TMR), on *in vitro* gas production (GP), CH_4_ and CO_2_ emissions, *in vitro* ruminal fermentation (total and individual short-chain fatty acids (SCFAs), nutrient degradability, metabolizable energy (ME), ruminal NH_3_-N, and partitioning factor (PF_24_) using rumen inoculum of sheep. It was hypothesized that at low levels of supplementation, the phytochemical constituents of the dried LS could increase ruminal microbial activities and growth (not measured), alter ruminal fermentation towards increasing ruminal propionate (C_3_) and acetate (C_2_), and increase nutrient degradability in sheep.

## 2. Materials and Methods

### 2.1. Ingredients and Treatments

A basal TMR containing [per kg of dry matter (DM)] 500 g of concentrate feed mixture, 400 g of berseem (*Trifolium alexandrinum*) hay, and 100 g of rice (*Oryza sativa*) straw was formulated and grounded. The incubated substrate or diet is the same as the control diet previously used by Kholif et al. [17,18,19]. The basal TMR or substrate (control treatment) was supplemented with graded levels of dried LS. There were thus five treatments comprising the following: (1) 1 g substrate + 0 g LS; (2) 1 g substrate + 0.005 g LS; (3) 1 g substrate + 0.01 g LS; (4) 1 g substrate + 0.015 g LS; and (5) 1 g of substrate + 0.02 g LS. Following the treatments, the substrate and the respective doses of LS were carefully weighed into the filter bags using a Luna Analytical Balance (LAB 124e, Adam Scales & Balances, Thetford, UK). They were subsequently thoroughly mixed together and placed inside fermenter bottles prior to incubation. The nutrient compositions of the LS and the TMR are detailed in Table 1.

Clean and dry LS were sourced from a local supplier in Egypt. Before use, the LS were ground and mixed thoroughly. To analyze the volatile compounds present in the LS, a GC-MS system (model 7890B from Agilent Technologies, Santa Clara, CA, USA) equipped with a flame ionization detector was employed at the Central Laboratories Network, National Research Centre, Dokki, Giza, Egypt. The analysis was conducted according to Qin et al. [20] with some modifications. The separation process utilized a Zebron ZB-FAME column (dimensions: 60 m × 0.25 mm internal diameter × 0.25 μm film thickness). The analysis was conducted using hydrogen as the carrier gas at a flow rate of 1.8 mL/min in split-1:50 mode, with an injection volume of 1 µL. The temperature program was set initially at 100 °C for 3 min, and subsequently increased at a rate of 2.5 °C per minute to 240 °C, where it was maintained for 10 min. The temperatures for the injector and the flame ionization detector (FID) were set at 250 °C and 285 °C, respectively. The concentration of individual identified phytochemicals was expressed as mg/100 g DM seeds.

### 2.2. In Vitro Fermentation and Biodegradation

The *in vitro* fermentation medium was prepared following the method outlined by Goering and Van Soest [21]. A reducing solution containing 2 mL of sodium sulfide was added to the buffer shortly before the addition of rumen fluid. Each 250 mL bottle contained a mix of 20 mL ruminal inoculum and 80 mL buffer solution.

The ruminal inoculum was sourced from three sheep at a local slaughterhouse in Cairo, Egypt. Prior to slaughter, the sheep were fed a diet of concentrates, berseem hay, and rice straw in a 500:400:100 ratio (DM basis) ad libitum, with free access to water. Rumen contents were collected following the standardized procedure for sampling, storage, and use of ruminal contents as recommended by Fortina et al. [22]. Sheep were fasted for 24 h before slaughtering. The time between the animal’s death and rumen fluid collection was under 10 min. Approximately 150–250 g of rumen contents was hand-sampled and squeezed into a plastic beaker through a colander, and the process was repeated until about 1000 mL of rumen fluid was collected. The rumen fluid was then filtered through a two-layered cheesecloth to remove large feed particles, and particulate material was squeezed to collect microbes attached to the feed. The initial pH of the inoculum was between 6.8 and 6.9. All the treatments were tested in two incubation runs (statistical replicates), each with three bottles (analytical replicates). In each run, two bottles with inoculum but without substrate (blanks) were included to establish baseline fermentation GP (5 treatments × 3 replicates × 2 incubation runs + 2 blank bottles).

Approximately 1 g (±10 mg) of TMR was weighed into filter bags (ANKOM F57; Ankom Technology, Macedon, NY, USA) and placed into 250 mL ANKOM bottles (AnkomRF Gas Production System) equipped with an automatic wireless *in vitro* GP module (Ankom Technology, Macedon, NY, USA) with pressure sensors. Lupin seeds were included at 0 (control), 0.5, 1, 1.5, and 2% of the TMR before incubation. Pressure was recorded every 10 min for 48 h, and cumulative pressure was calculated from these values. Gas pressure was converted into volume (mL) at standard pressure and temperature, and the gas volume in the blank units was subtracted to obtain net GP. At 2, 4, 6, 8, 10, 12, 24, 36, and 48 h of incubation, 5 mL gas samples were taken from the sampling vent and analyzed using a Gas-Pro detector (Gas Analyzer CROWCON Model Tetra3, Abingdon, UK) to measure CH_4_ and CO_2_ concentrations.

### 2.3. Sampling and Analysis of Fermentation Variables

After 48 h of incubation, fermentation was halted by chilling the bottles on ice for 5 min, followed by immediate pH measurement using a pH meter (Thermo Scientific, Orion Star™ A121, Beverly, MA, USA). The ANKOM F57 filter bags were dried in a forced air oven at 55 °C for 48 h. The degradation of dry matter (*d*DM), neutral detergent fiber (*d*NDF), and acid detergent fiber (*d*ADF) was determined by subtracting the weight of the dried residue from the initial weight of the dried substrate. Total gas and CH_4_ production were normalized to *d*DM, *d*NDF, and *d*ADF at the 48 h mark of incubation.

Samples of the fermented fluid supernatant (5 mL) from each bottle were collected in glass tubes for analyzing NH_3_-N concentration, as well as total and individual SCFAs. A 3 mL subsample, was preserved with 3 mL of 0.2 M hydrochloric acid solution to measure NH_3_-N concentration according to AOAC [23] guidelines. 

A 0.8 mL aliquot was mixed with 0.2 mL of metaphosphoric acid solution (250 g/L) for SCFA analysis using steam distillation and titration.

### 2.4. Chemical Analysis

The LS and TMR samples were subjected to ash analysis by burning them in a muffle furnace at 550 °C for 12 h (method ID 942.05), CP analysis using the Kjeldahl method (method ID 954.01), and ether extract (EE) determination using diethyl ether in Soxhlet extractors (method ID 920.39) following AOAC methods [23]. Neutral detergent fiber (NDF) content was determined without alpha amylase but with sodium sulfite, following the Van Soest et al. [24] procedure. Acid detergent fiber (ADF) content was analyzed according to AOAC [23] (method ID 973.18) and expressed without residual ash. Non-structural carbohydrate, cellulose, hemicellulose, and organic matter (OM) concentrations were calculated.

### 2.5. Calculations and Statistical Analyses

To analyze the kinetics of GP, CH_4_, and CO_2_, the data on total GP, CH_4_, and CO_2_ (measured in mL/g of dry matter) were fitted using the NLIN procedure in SAS (version 9.4, SAS Inst., Inc., Cary, NC, USA), following the model proposed by France et al. [25]: y = A × [1 − e^−c (t−Lag)^], where y represents the volume of total GP, CH_4_, or CO_2_ production at time t (in hours); A is the asymptotic GP, CH_4_, or CO_2_ (measured in mL/g DM); c is the fractional rate of GP, CH_4_, or CO_2_ (per hour), and Lag (/h) is the discrete lag time before any gas, CH_4_, or CO_2_ release occurs.

The partitioning factor at 24 h of incubation (i.e., PF_24_; in mg of dry matter: mL of gas production) was also determined [26]. The gas volume produced (in mL per 200 mg DM) at 24 h of incubation (GY_24_) was computed. Metabolizable energy (ME) was calculated following the method by Menke et al. [27], and microbial crude protein (MCP) production was estimated using the approach of Blümmel et al. [26].

Statistical analysis was performed using the GLM procedure in SAS, employing a completely randomized design with the model: Y_ij_ = μ + L_i_ + ε_ij_, where Y_ij_ represents the observation, μ is the population mean, L_i_ is the effect of LS supplementation level, and ε_ij_ is the residual error. Data of each of the two runs of the same sample of the substrate were averaged prior to statistical analysis. Mean values of each individual run (two runs) were used as the experimental unit. Linear and quadratic contrasts were applied to assess the responses at different levels of LS supplementation.

## 3. Results

### 3.1. Lupin Seeds

Lupin seed contained 3.27% essential oils (DM basis), with five volatile compounds identified: α- and β- pinene, eucalyptol, camphor, and trans-caryophyllene (Table 2). They were all C_10_ except for trans-caryophyllene which was a C_15_ compound. Eucalyptol was the most prominent (86.7%) bioactive compound in the LS while the least was trans-caryophyllene.

### 3.2. Biogas Production

**Figure 1:**
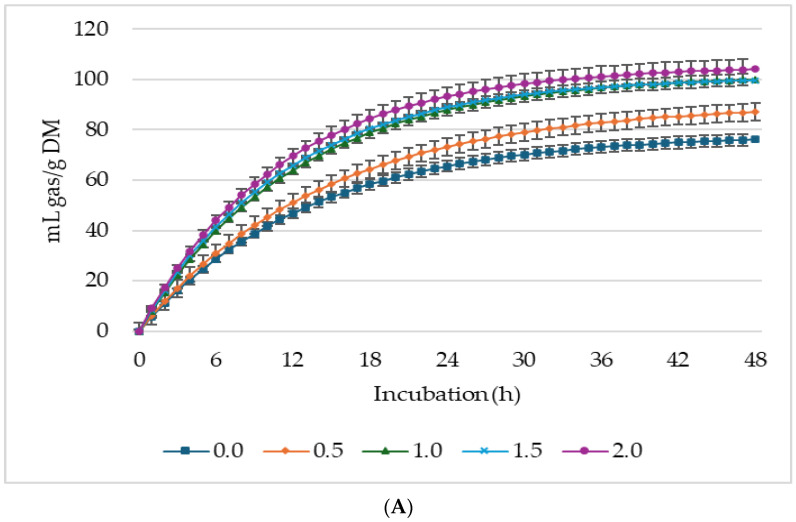

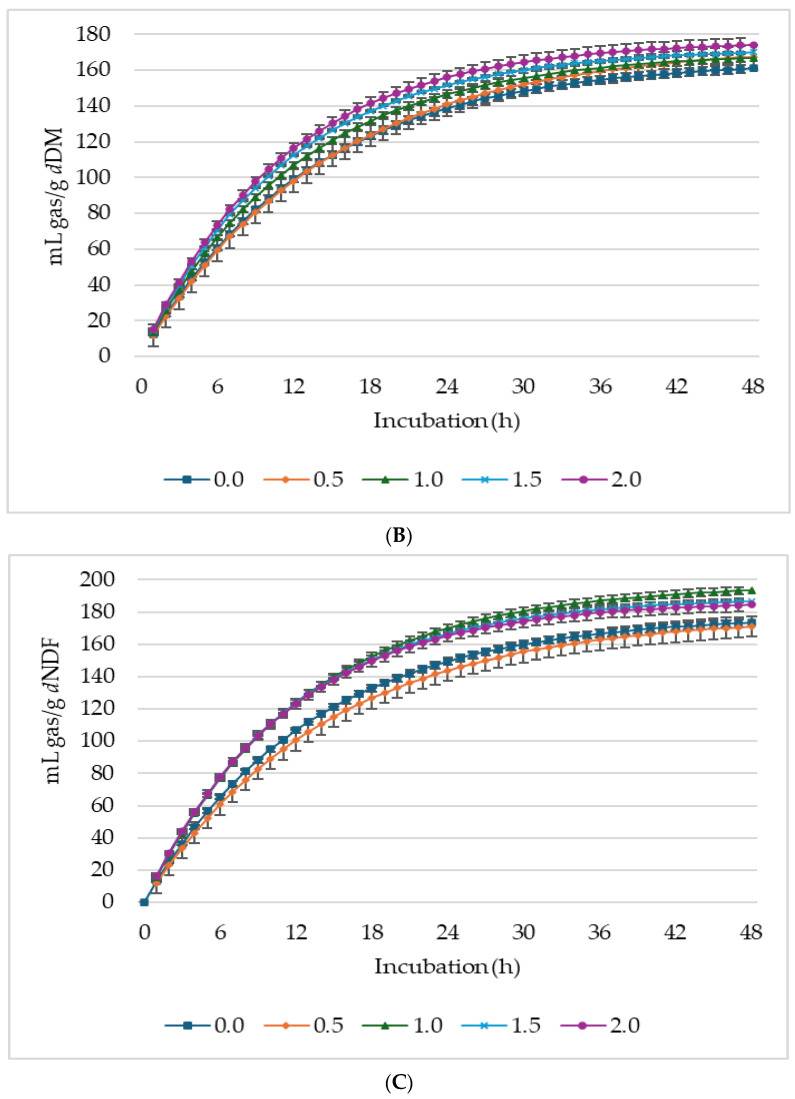

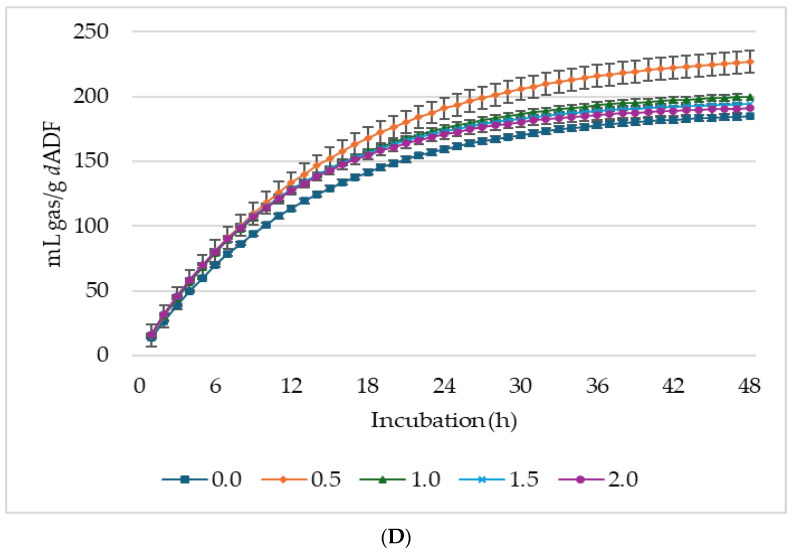
*In vitro* ruminal gas production: mL/g incubated DM (**A**), mL/g degradable DM (**B**), mL/g degradable NDF (**C**), mL/g degradable ADF (**D**) of a total mixed ration supplemented with different levels of lupin seeds.

**Figure 2:**
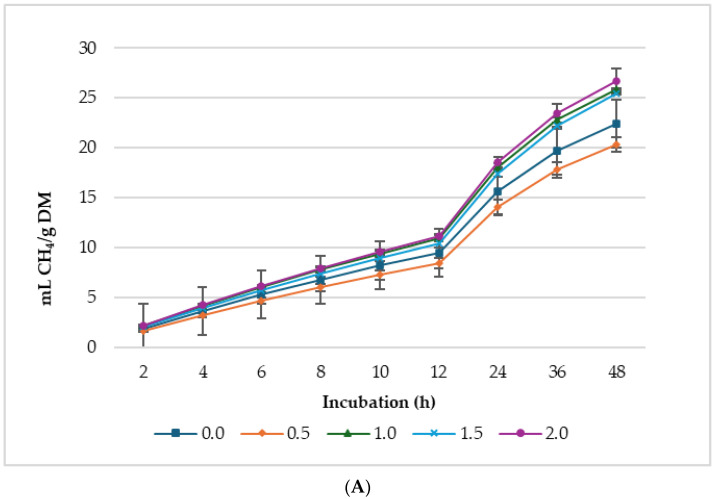

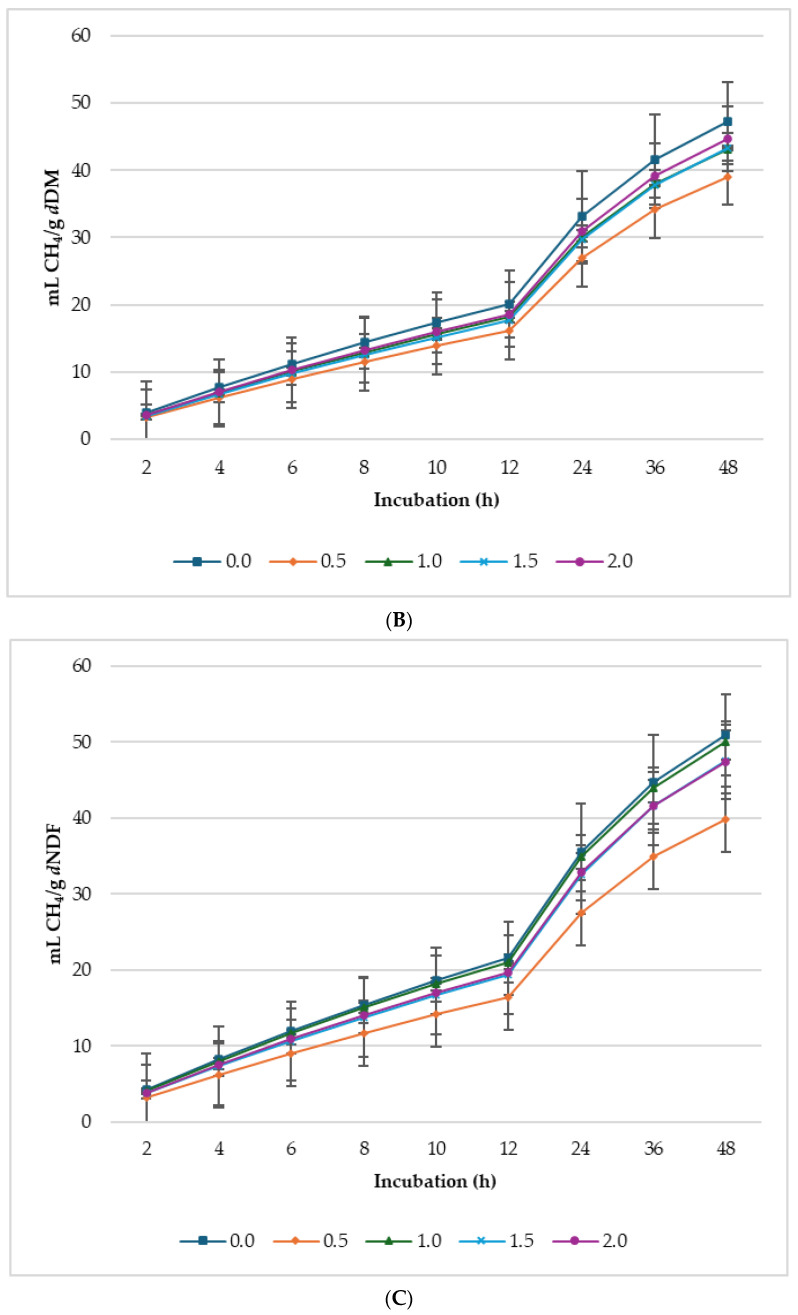

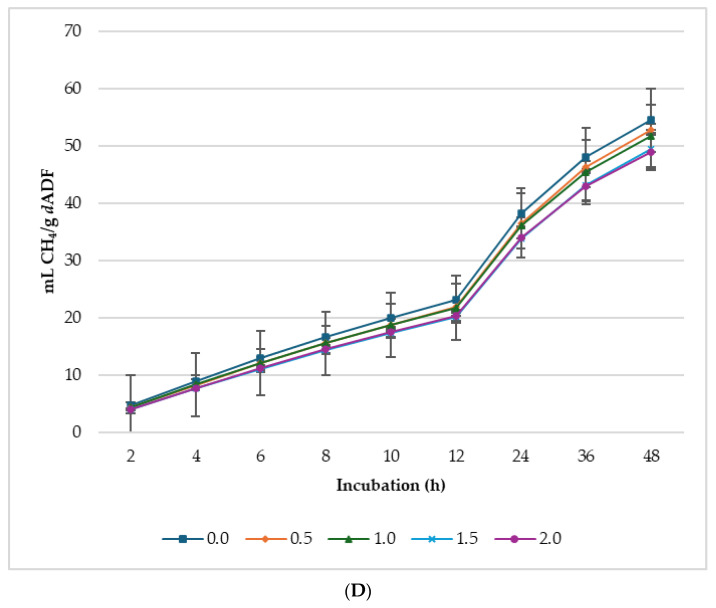
*In vitro* ruminal methane (CH_4_) production: mL/g incubated DM (**A**), mL/g degradable DM (**B**), mL/g degradable NDF (**C**), mL/g degradable ADF (**D**) of a total mixed ration supplemented with different levels of lupin seeds.

**Figure 3:**
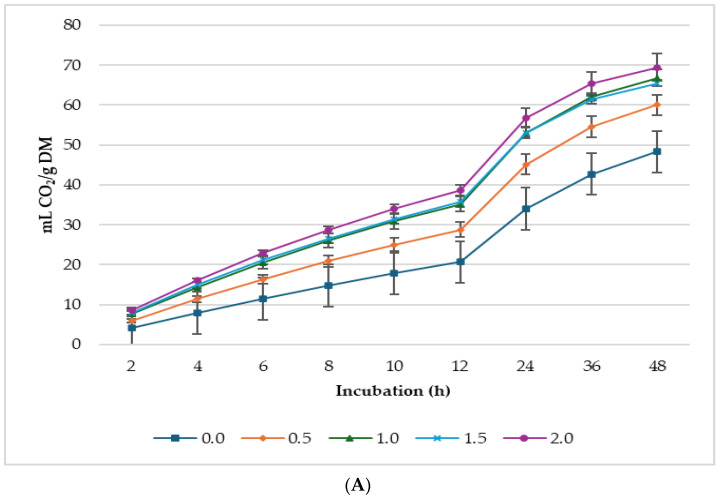

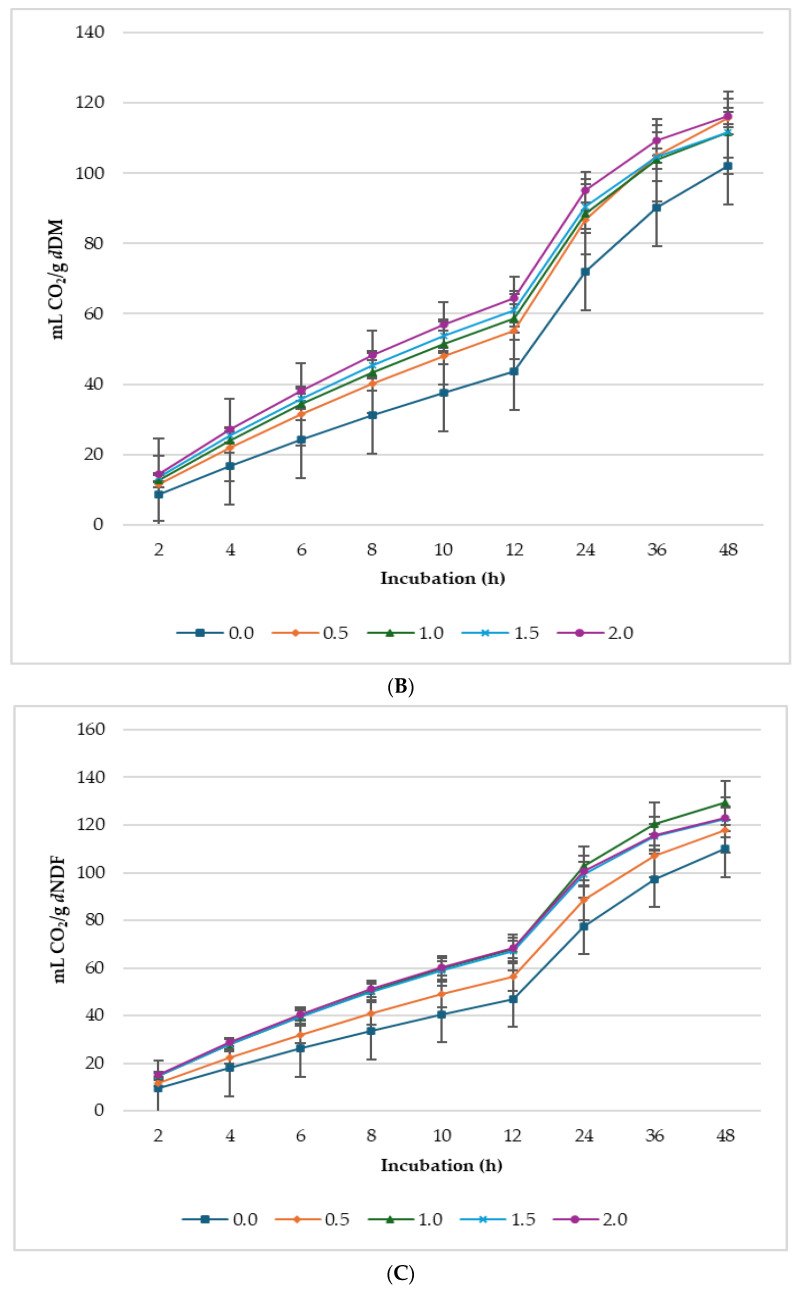

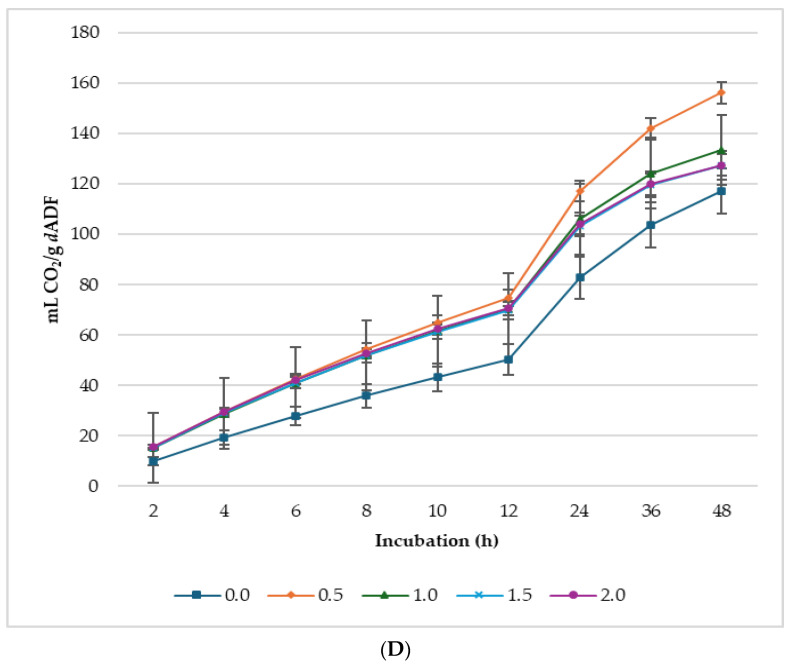
*In vitro* ruminal carbon dioxide (CO_2_) production: mL/g incubated DM (**A**), mL/g degradable DM (**B**), mL/g degradable NDF (**C**), mL/g degradable ADF (**D**) of a total mixed ration supplemented with different levels of lupin seeds.

Figure 1, Figure 2 and Figure 3 show GP, CH_4_, and CO_2_ production per gram DM, *d*DM, *d*NDF, and *d*ADF. Lupin seed at 2% supplementation level of the incubated diet had the highest GP (mL/g DM; mL/g *d*DM) among the treatments. Whereas GP (mL/g *d*NDF) was greater in 1.0% LS treatment, 0.5% lupin diet produced the lowest gas. Conversely, 0.5% lupin produced the greatest gas for every gram of *d*ADF. It is worth noting that the concentrations of CH_4_ and CO_2_ (Figure 1 and Figure 2) did not appear to follow the same pattern as GP. This discrepancy arises because GP was measured every 10 min, whereas CH_4_ and CO_2_ were initially measured every 2 h until 12 h of incubation after which they were measured every 12 h (i.e., at 24, 36, and 48 h of incubation).

Supplementation with LS significantly impacted the asymptotic GP, CH_4_, and CO_2_ production (Table 3). The asymptotic GP showed both linear (*p* < 0.001) and quadratic (*p* = 0.002) increases, whereas the hourly rate of GP exhibited a linear increase (*p* = 0.001) in response to increasing LS levels. Lag time was linearly (*p* = 0.021) reduced with increasing LS in the diet. Asymptotic CH_4_ production linearly (*p* = 0.002) increased with increasing level of LS, and the lag time, i.e., the time taken for initial GP, was prolonged by the supplementation. Despite the increase in asymptotic CH_4_ production of the treatments, the proportion of CH_4_ in the total biogas was lower (*p* < 0.05) than the control, and 0.5% LS had the lowest proportion of CH_4_ in total biogas production. The control treatment produced the highest gas for every gram of *d*NDF and *d*ADF, while 0.5% produced the lowest (Figure 3).

Similarly, asymptotic CO_2_ and the rate of GP per hour linearly (*p* ≤ 0.001) increased with increasing levels of LS. The proportion of CO_2_ in the total GP was higher (*p* = 0.027) in the treatments than in the control, with 0.5% lupin having the highest proportion of CO_2_ in total biogas produced. In Figure 3, 1.0% lupin produced the highest volume of CO_2_ for every gram of *d*NDF, 0.5% produced the highest volume of CO_2_ for every gram of *d*ADF, and the diet without lupin produced the lowest in both cases.

### 3.3. Degradability and Fermentation

Table 4 presents the rumen fermentation profile of diets with increasing levels of LS supplementation. *d*DM increased (*p* < 0.001) with increasing levels of LS in the diet. Furthermore, *d*NDF and *d*ADF increased (*p* < 0.01) with increasing levels of LS, with the diet containing 2.0% LS being the most degraded. However, *d*ADF was lowest (*p* < 0.001) for 0.5% LS among the treatments.

Total SCFAs, C_2_, and C_3_ linearly (*p* < 0.01) increased with the level of LS in the diet. However, C_2_:C_3_ ratio linearly (*p* = 0.015) reduced as the LS level in the diet increased. Although butyrate (C_4_) was not (*p* = 0.155) influenced by LS addition to the diet, it linearly (*p* = 0.025) increased with increasing LS levels.

Rumen pH decreased (*p* = 0.036) with increasing levels of LS. The decrease was linear (*p* = 0.015) except for the diet containing 0.5% of lupin, which had a higher pH than the control. While ruminal NH_3_-N linearly (*p* = 0.008) decreased, ME linearly and quadratically (*p* < 0.001) increased with increasing levels of LS. The partitioning factor was not (*p* = 0.093) affected by LS, but there was a linear (*p* = 0.009) decrease in the grams of feed degraded per volume of GP. Microbial crude protein (MCP) was linearly (*p* = 0.001) and quadratically (*p* = 0.018) affected by treatment, with diet containing 1.0% LS having the highest MCP. However, LS-supplemented diets had higher (*p* = 0.005) MCP compared to the control. Gas yield at 24 h (GY_24_) linearly (*p* = 0.006) increased with the doses of LS.

## 4. Discussion

### 4.1. Lupin Seeds

Bioactive components of a plant can serve as either antinutritional or nutraceutical agents, influencing nutrient absorption and availability. Currently, there are limited studies on the bioactive components of LS. In the present study, five compounds were identified, with four containing C_10_ carbon and one containing C_15_ carbon. Compounds such as α- and *β*-pinene and camphor had 16 hydrogen atoms (H_16_), while others like eucalyptol and trans-caryophyllene had 18 (H_18_) and 24 (H_24_) hydrogen atoms, respectively. Phytochemical constituents are known to possess antimicrobial, antioxidant, and anti-inflammatory properties [8]. Lupin seeds in this study contained a higher proportion of eucalyptol compared to the other four compounds combined. Therefore, eucalyptol, with the concentration of 2835 mg/100 g DM, was the major bioactive compound, accounting for 86.7% of total phytochemicals in the LS.

Previous phytochemical studies on lupin focused on alkaloids [28,29], without considering other compounds evaluated in the current study. The concentration of eucalyptol, which has the ability to positively or negatively influence ruminal microbes [30], in LS is similar to that found in peppermint leaves. The presence of phytochemicals in LS highlights their potential as a functional feed additive. These phytochemicals can positively influence ruminal microflora activity and growth, leading to improved nutrient digestion and animal performance. Different responses with the evaluated levels of LS in different experiments may be mainly due to different concentrations of phytochemicals.

### 4.2. Gas Production

Generally, GP is used to measure the rate of fermentation in anaerobic conditions, and a greater rate of fermentation can lead to gases accumulation. Though gas accumulation is of paramount importance, the proportion of the gases produced in total GP is also of interest. This is because sometimes the proportion of environmentally unhealthy gases may comprise a larger portion of the total gases. In the present experiment, the rise in asymptotic GP of the lupin supplemented diets could stem from its contents of phytochemicals which perhaps fostered microbial activities. It appears that certain phytochemical constituents of the LS enhanced microbial proliferation and activity, resulting in an increased fermentation rate and microbial protein synthesis, ME content, and fiber digestion [31]. The prevalence of β-galactan in lupins might positively impact rumen microbial populations [14]. The accelerated rate of GP per hour and reduced lag time for GP suggest faster adaptability of rumen microbes to lupin-supplemented diets. Although lupin seeds are known to contain quinolizidine alkaloids, which may be unfavorable to animals, the improved fermentation (increased GP, nutrient degradability, and SCFAs) may be because the variety of lupin used in the present experiment had reduced alkaloid contents [32,33]. 

Methane production accounts for substantial dietary energy losses, which can impact ruminant productivity significantly. Additionally, enteric CH_4_ emissions from ruminants contribute to approximately 17% of global greenhouse gas emissions [2]. Although CH_4_ from ruminants is biogenic in nature, the short-term environmental impact calls for the need to reduce its emission. Though the volumes of CH_4_ produced were higher for the LS supplemented diets than for the control diet except for the diet containing a 0.5% level of LS, a closer look at the proportion of CH_4_ in the total biogas shows a trade-off in that the proportion of methane in the biogas decreased. It can be seen that all the treatments had lower proportions of CH_4_ for every milliliter of total biogas. This result can be plausibly attributed to the antimicrobial property of the phytoconstituents of LS, which perhaps reduced ruminal microbes, such as protozoa and methanogens, responsible for methanogenesis. Bryszak et al. [34] showed that dietary inclusion of lupin, particularly *Lupinus angustifolius* seed meal at 80 and 100 g/kg in a complete diet containing a concentrate-to-forage ratio of 448:552, reduced total bacteria, archaea, and total protozoa in an *in vitro* experiment. Methanogenesis arises from the interactions between hydrogen-producing microorganisms (bacteria, protozoa, and fungi) and methane-consuming microorganisms (methanogens) in the rumen, maintaining environmental homeostasis [34]. Thus, the reduced CH_4_ output could be due to lower bacterial and methanogen activities or a shift in methanogen populations towards less efficient methane-producing species [35]. Notably, the lupin fatty acid profile includes C12:0 and C14:0 acids, which exhibit antimicrobial effects against protozoa [36,37]. Bryszak et al. [34] demonstrated that feeding lupin seeds at 2 kg/day to cows fed a total mixed diet containing concentrate and forage at 462:538 reduced the total count of methanogens, including Methanobacteriales and Methanomicrobiales classes, leading to decreased methanogenesis. Methanogens possess the capability to convert CO_2_ to CH_4_ by utilizing various substrates during fermentation [38]. Unfortunately, we did not measure H_2_ concentration to elucidate the proper picture of how lupin affected CH_4_ emissions. Nevertheless, it was conjectured that they probably achieved this by positioning themselves on the outer layers of the biofilm, allowing them to access H_2_ diffusing from the carbohydrate fermentation site [39]. Subsequently, they possibly combined H_2_ with CO_2_ to generate CH_4_. A further look at the CO_2_ showed that there was a reduction in the combination of CO_2_ and H_2_ to form CH_4_. It is pertinent to say that the 0.5% LS supplementation level, with the lowest proportion of CH_4_, had the highest proportion of CO_2_. This suggests that as the metabolic pathways for the formation of CH_4_ were hindered, the materials for CH_4_ formation, such as CO_2_ and H_2_, became the alternatives which were eructated.

### 4.3. Degradability and Fermentation

Dry matter degradability (i.e., *d*DM) of a feed or forage is a key indicator of its nutritional quality and potential to provide energy to animals [40,41]. The *d*DM, along with *d*ADF and *d*NDF, reflects the extent to which different components of a feed are broken down or degraded by rumen microorganisms. The increased *d*DM, *d*ADF, and *d*NDF with increasing levels of lupin indicates the importance of defining the optimal level (dose) of LS supplementation. This may be attributed to the inherent characteristics of lupin, especially phytochemicals which possibly enhanced microbial proliferation (growth and activity), leading to the improved colonization and degradation of the diet [42]. The inherent phytochemical components of the LS make it an appealing functional supplement in ruminant diets. It is pertinent to say that the increases in DM, NDF, and ADF degradability were not due to the nutritive attribute of the LS but rather the functional properties or phytogenic compounds, which were likely responsible for enhanced microbial activities. Thus, the phytochemicals of the LS likely aided digestibility, which increased with higher doses or levels [8,9]. The increased degradability of *d*DM, *d*ADF, and *d*NDF is beneficial as it connotes the potential of LS to enhance nutrients and energy availability to animals.

Lupin seed supplementation increased C_2_ and C_3_ concentrations. Each of C_2_, C_3_, and C_4_ are SCFAs produced during rumen fermentation in ruminants. The increased C_3_ is important because it serves as a major precursor for glucose synthesis in the liver through gluconeogenesis, helps to regulate blood glucose levels, and contributes to overall energy metabolism, which is important for lactating animals to meet their energy requirements for milk production [43]. Moreover, the increased C_2_ production is also an important result, especially in dairy animals, because it serves as a precursor for fatty acid synthesis, particularly in adipose tissue and milk [44,45]. Gas production is directly linked to SCFA levels, with higher GP indicating higher SCFA levels [46,47]. Consequently, increased SCFA and ME values are associated with higher GP and digestibility. The levels of SCFAs reflect energy availability and can contribute up to 80% of the animal’s daily energy requirement [48], showing a direct correlation with ME and degradable OM [27]. Additionally, SCFA levels can be used to establish connections between feed composition, production parameters, and the net energy value of feeds [49]. The higher SCFA concentration of the treatments is obviously the result of increased fermentability and nutrient availability for ruminal microbial growth and activity for enhanced degradability [50]. The increased SCFAs, C_2_, and C_3_ of the LS treatments align with the finding of Um et al. [10], who reported that diets containing lupin cake at 3, 6, and 9% showed higher production of propionate, butyrate, and total volatile fatty acids in an *in vitro* experiment. The results, however, contradict the work of Bryszak et al. [34], where increased LS meal decreased total and individual SCFAs. This variation in results may be attributed to the lupin varieties and doses used in different studies, since different varieties have different phytochemicals (type and concentrations) [32,33]. The lower C_2_:C_3_ ratio with increasing levels of lupin is nutritionally beneficial as it indicates that LSs favor an increase in the production of gluconeogenic acids. The lower C_2_:C_3_ ratio may also contribute to reduced CH_4_ production [43] and a decrease in the activity of cellulolytic bacteria such as *R*. *albus* and *F*. *succinogenes* [51]. The linear increase in the C_4_ acid with increasing levels of lupin suggests that lupin seeds, a functional feed additive, can support both dairy and beef production systems and can be used in ruminant nutrition for altering ruminal fermentation and affecting the production of individual SCFAs.

Rumen pH is a crucial measure that reflects the balance between acids and bases in a solution, playing a central role in understanding nutritional diseases and nutrient digestion in ruminants [52]. Recent studies suggest that diet type may not be the sole reason for fluctuations or depressions in rumen pH. Rather, the increase in dissolved CO_2_ (*d*CO_2_) concentration in rumen liquor has been identified as a key factor [53]. While rumen *d*CO_2_ concentrations are typically low and stable, modern feeding practices can lead to CO_2_ holdup, defined as a decrease in CO_2_ fugacity due to changes in rumen liquor’s physicochemical properties. This gas holdup can contribute to elevated rumen *d*CO_2_ concentrations, leading to a subsequent decline in pH [53]. In the present experiment, ruminal pH decreased with increasing levels of LS, but obtained values were within the normal pH reported by Kamra [54] and Ososanya et al. [55] for optimal rumen function. The decreased ruminal pH with increasing LS levels indicates rapid fermentation of the readily fermentable carbohydrate content of the diets to volatile fatty acids [56,57], indicating the importance of defining the optimal level of LS supplementation. Ruminal pH is related to SCFA production, which is associated with OM degradability, primarily carbohydrates (both readily degradable and structural carbohydrates) degradability in the rumen environment. Therefore, a lower pH, as a consequence of greater DM degradability, is expected. As previously mentioned, the increases in DM digestibility were not due to the nutrients in the LS but rather the changes in microbial activity as a consequence of the phytogenic compounds. A positive correlation between readily fermentable carbohydrate and volatile fatty acids, and a negative correlative between pH and volatile fatty acids [58] have been reported.

Ammonia-N plays a crucial role in the digestion and metabolism of feed materials by rumen microorganisms. Ammonia-N is a byproduct of the breakdown of proteinaceous compounds such as plant proteins and microbial proteins. It is a key step in protein degradation within the rumen and serves as a crucial nitrogen source for rumen microbes. Microbes assimilate NH_3_-N to synthesize microbial protein, which is then utilized by the animal upon microbial cell death or passage from the rumen to provide a significant portion of the dietary protein requirements for ruminants [59]. The decrease in NH_3_-N with increasing levels of lupin implies efficient N utilization. Ammonia serves as a N source used by rumen bacteria to meet their nitrogen requirement for body protein synthesis. However, ciliate protozoa do not utilize ammonia. The recycling of bacterial N in the rumen increases in the presence of ciliate protozoa, and the number of ruminal bacteria capable of utilizing NH_3_-N decreases with increased ruminal breakdown of dietary protein. Hence, the decrease in NH_3_-N concentration could be partially attributed to lupin’s ability to reduce protozoa numbers [34] and decrease hyper-ammonia producing bacteria due to its phytochemical constituents [8].

Inclusion of up to 6% lupin flakes in the diet of cows improved the efficiency of N utilization and decreased ruminal NH_3_-N concentration [60]. The improved efficiency of N utilization reduces NH_3_-N concentration, which is used by rumen microbes for their proliferation, resulting in increased MCP. This perhaps explains the reason for the increased MCP and reduced rumen NH_3_-N concentration of the LS treatments compared to the control. Microbial crude protein supplies amino acids to the small intestine for absorption, underscoring its usefulness to ruminant animals. The synthesis of MCP depends on various factors such as the sources of carbohydrates and proteins, the synchronization of rumen functions, rumen microbial recycling, and the presence of anti-nutrients in consumed feeds [61]. Moderate quantities of carbohydrates that are readily fermentable in a diet can sometimes increase microbial protein synthesis [62]. This increase occurs due to the enhanced availability of substrates and the growth rate of bacteria associated with the liquid phase of digestion [61,62]. Likewise, there is an increment in the enhancement of degradable OM. Therefore, it seems that LSs show potential in eliminating some microorganisms that are not beneficial in the rumen of ruminant livestock. Simultaneously, they create a conducive environment for the growth and proliferation of beneficial microbes, thereby increasing feed digestibility. Phytochemical-rich LS enhanced the ME, PF_24_, and MCP levels, indicating an ideal combination of energy and protein, leading to increased microbial protein synthesis and PF_24_ [18]. The phytochemicals in LS may interfere with the biosynthesis of aromatic amino acids, as synthesis pathways are linked through phytochemicals from the seeds [19]. Results from increased MCP indicate that most of the NH_3_-N and SCFAs were used for MCP synthesis [17,18,19].

Gas yield for every gram of DM digested after 24 h (GY_24_) shows more gases were produced per gram of DM digested as LS increased. The higher GY_24_ of the LS supplemented diets thus indicates an enhanced incubation environment and fermentability [19]. 

## 5. Conclusions

Lupin seeds contained certain bioactive compounds such as α- and β-pinene, eucalyptol, camphor, and trans-caryophyllene, which can significantly modulate rumen fermentation and GP. Lupin seed supplementation of ruminant diets led to dose-dependent increases in GP, with higher levels of lupin seeds resulting in increased asymptotic gas, CH_4_, and CO_2_ production. Interestingly, despite the increase in CH_4_ production, the proportion of CH_4_ in total biogas was reduced with lupin supplementation compared to the control treatment, suggesting a potential mitigation effect on CH_4_ emissions. Furthermore, lupin seed supplementation enhanced *d*DM, *d*ADF, and *d*NDF, indicating improved nutrient utilization. The increased SCFAs and ME with lupin seed treatments suggest higher energy availability. Overall, the findings of this study underscore the potential of lupin seeds supplementation of a total mixed ration at 2.0% (DM basis) for ruminants to improve ruminal fermentation efficiency and nutrient utilization, and mitigate CH_4_ emissions. Further research should be conducted in vivo to validate the findings of the current study.

## Figures and Tables

**Table 1:** Chemical composition of lupin seeds and incubated diet (g/kg DM).

	Lupin Seeds	CFM ^1^	Berseem Hay	Rice Straw	Diet ^2,3^
Dry matter	927	903	890	940	893
Organic matter	965	923	884	851	819
Crude protein	200	165	128	42	136
Ether extract	144	47	54	19	62
Nonstructural carbohydrates	429	414	224	166	359
Neutral detergent fiber	192	297	478	624	379
Acid detergent fiber	179	175	381	394	240

^1^ The concentrate feed mixture (CFM) used in the study consisted of the following components per kilogram of dry matter (DM): 170 g of soybean meal, 395 g of wheat bran, 395 g of maize, 20 g of limestone, 10 g of a vitamins and minerals mixture, and 10 g of salt. ^2^ The diets used in the experiment contained, per kilogram of DM, the following: 500 g of the concentrate mixture, 400 g of berseem hay (*Trifolium alexandrinum*), and 100 g of rice straw (*Oryza sativa*). ^3^ The treatments were as follows: (1) 1 g substrate + 0 g LS; (2) 1 g substrate + 0.005 g LS; (3) 1 g substrate + 0.01 g LS; (4) 1 g substrate + 0.015 g LS; and (5) 1 g of substrate + 0.02 g LS. Following the treatments, the substrate and the respective doses of LS were carefully weighed into the filter bags using a Luna Analytical Balance (LAB 124e, Adam Scales & Balances, Thetford, UK).

**Table 2:** Volatile compounds in lupin seeds identified by GC-MS analysis.

Peak	Compound ^1^	Formula	RT ^2^	Concentration ^3^ (%)	Concentration (mg/100 g DM)
1	α-Pinene	C_10_H_16_	3.702	4.33	142
2	β-Pinene	C_10_H_16_	4.771	2.81	92
3	Eucalyptol	C_10_H_18_O	6.738	86.7	2835
4	Camphor	C_10_H_16_O	9.942	3.71	121
5	trans-Caryophyllene	C_15_H_24_	15.221	2.48	81

^1^ Identification was conducted using authentic standards, spectra from the National Institute of Standards and Technology (NIST) library, and relevant literature sources. ^2^ RT, retention time (min). ^3^ The concentration was determined based on the total areas of the identified peaks.

**Table 3:** The kinetics of *in vitro* rumen gas production (GP), methane (CH_4_), and carbon dioxide (CO_2_) were influenced by increasing levels of lupin seeds.

	GP Parameters ^1^				CH_4_ Parameters ^2^					CO_2_ Parameters ^3^			
Level (%, DM)	*A*	*c*	Lag		*A*	*c*	Lag	% ^4^		*A*	*c*	Lag	% ^4^
0	78.1 ^c^	0.076 ^bc^	1.55 ^ab^		28.6 ^ab^	0.035	1.42 ^b^	29.3 ^a^		58.8 ^b^	0.036 ^c^	2.47	63.5 ^b^
0.5	90.5 ^b^	0.070 ^c^	1.61 ^a^		25.5 ^b^	0.033	1.44 ^b^	23.3 ^b^		67.8 ^ab^	0.047 ^bc^	2.22	68.9 ^a^
1	102.0 ^a^	0.082 ^ab^	1.59 ^a^		31.8 ^a^	0.035	1.49 ^ab^	25.9 ^ab^		71.6 ^a^	0.056 ^ab^	2.25	66.7 ^ab^
1.5	101.2 ^a^	0.088 ^a^	1.47 ^b^		32.3 ^a^	0.032	1.57 ^ab^	25.5 ^ab^		69.3 ^a^	0.061 ^a^	2.14	65.6 ^ab^
2	105.5 ^a^	0.090 ^a^	1.34 ^c^		33.2 ^a^	0.034	1.68 ^a^	25.6 ^ab^		73.0 ^a^	0.063 ^a^	2.19	66.7 ^ab^
SEM	1.82	0.002	0.06		1.03	0.0032	0.05	0.84		2.11	0.0029	0.17	0.94
*p* value													
Treatment	<0.001	0.006	0.033		0.002	0.976	0.014	0.008		0.006	0.004	0.716	0.027
Linear	<0.001	0.001	0.021		0.006	0.823	0.001	0.080		0.001	<0.001	0.277	0.326
Quadratic	0.002	0.328	0.082		0.564	0.893	0.280	0.015		0.061	0.076	0.485	0.053

Means in the same column with different superscripts differ (*p* < 0.05). The *p*-value represents the observed significance level of the F-test for the treatment effect, while SEM stands for the standard error of the mean.^1^ GP parameters: *A* is the asymptotic GP (mL/g DM), *c* is the rate of GP (/h), Lag is the initial delay before GP begin (h).^2^ Methane (CH_4_) production parameters: *A* is the asymptotic CH_4_ production (ml/g DM), *c* is the rate of CH_4_ production (/h), Lag is the initial delay before CH_4_ production begins (h). ^3^ Carbon dioxide (CO_2_) production parameters: *A* is the asymptotic CO_2_ production (ml/g DM), *c* is the rate of CO_2_ production (/h), Lag is the initial delay before CO_2_ production begins (h). ^4^ The proportion at the end of incubation (48 h).

**Table 4:** *In vitro* rumen fermentation profile of diet with increasing levels of lupin seeds.

	Degradability (g/kg DM) ^1^				SCFA (mmol/g DM ) ^2^						Fermentation ^3^					
Level (%, DM)	*d*DM	*d*NDF	*d*ADF		Total	C_2_	C_3_	C_2_:C_3_	C_4_		pH	NH_3_-N	ME	PF_24_	MCP	GY_24_
0	473 ^b^	439 ^b^	412 ^b^		23.4 ^b^	11.43 ^b^	7.90 ^b^	1.47 ^b^	4.08		6.27 ^a^	10.43 ^a^	4.68 ^c^	7.22	328 ^b^	139 ^b^
0.5	521 ^b^	512 ^ab^	386 ^b^		24.7 ^b^	12.83 ^ab^	8.11 ^b^	1.59 ^a^	3.81		6.30 ^a^	10.27 ^a^	5.33 ^b^	7.13	360 ^ab^	141 ^b^
1	599 ^a^	517 ^a^	501 ^a^		26.5 ^ab^	13.11 ^ab^	8.94 ^ab^	1.47 ^b^	4.45		6.13 ^b^	10.00 ^ab^	5.73 ^a^	6.82	406 ^a^	147 ^ab^
1.5	587 ^a^	534 ^a^	514 ^a^		28.8 ^a^	13.38 ^ab^	10.39 ^a^	1.29 ^c^	5.08		6.13 ^b^	9.63 ^b^	5.76 ^a^	6.60	391 ^a^	152 ^a^
2	598 ^a^	566 ^a^	546 ^a^		29.5 ^a^	13.94 ^a^	10.38 ^a^	1.34 ^c^	5.19		6.17 ^b^	9.43 ^b^	5.88 ^a^	6.41	393 ^a^	156 ^a^
SEM	11.7	16.2	13.0		0.84	0.467	0.406	0.100	0.418		0.039	0.201	0.051	0.208	11.5	3.8
*p* value																
Treatment	<0.001	0.003	<0.001		0.002	0.034	0.003	0.029	0.155		0.036	0.045	<0.001	0.093	0.005	0.047
Linear	<0.001	0.003	<0.001		0.001	0.004	0.002	0.015	0.025		0.015	0.008	<0.001	0.009	0.001	0.006
Quadratic	0.004	0.260	0.780		0.817	0.355	0.900	0.630	0.640		0.285	0.863	<0.001	0.890	0.018	0.815

Means in the same column with different superscripts differ (*p* < 0.05). The *p*-value represents the observed significance level of the F-test for the treatment effect, while SEM stands for the standard error of the mean. ^1^ *d*DM stands for dry matter degradability, *d*NDF represents neutral detergent fiber degradability, and *d*ADF indicates acid detergent fiber degradability (measured in g/kg of incubated material). ^2^ SCFA stands for short-chain fatty acid, where C_2_ represents acetate, C_3_ denotes propionate, and C_4_ indicates butyrate. ^3^ NH_3_-N refers to ammonia-N (measured in mg/g of dry matter), GY_24_ represents gas yield at 24 h (measured in mL of gas per gram of dry matter), ME stands for metabolizable energy (measured in MJ/kg of dry matter), PF_24_ indicates the partitioning factor at 24 h of incubation (measured in mg of degradable dry matter: mL of gas), and MCP denotes microbial crude protein production (measured in mg/g of dry matter).

## Data Availability

The original contributions presented in the study are included in the article, further inquiries can be directed to the corresponding authors.

## References

[1] Johnson K.A., Johnson D.E. (1995). Methane Emissions from Cattle. J. Anim. Sci..

[2] Knapp J.R., Laur G.L., Vadas P.A., Weiss W.P., Tricarico J.M. (2014). Invited Review: Enteric Methane in Dairy Cattle Production: Quantifying the Opportunities and Impact of Reducing Emissions. J. Dairy Sci..

[3] Dijkstra D.S., Linnemann A.R., Van Boekel T.A.J.S. (2003). Towards Sustainable Production of Protein-Rich Foods: Appraisal of Eight Crops for Western Europe. PART II: Analysis of the Technological Aspects of the Production Chain. Crit. Rev. Food Sci. Nutr..

[4] van Barneveld R.J. (1999). Understanding the Nutritional Chemistry of Lupin (*Lupinus* spp.) Seed to Improve Livestock Production Efficiency. Nutr. Res. Rev..

[5] Johnson S.K., Clements J., Villarino C.B.J., Coorey R., Taylor J.R.N., Awika J.M. (2017). Lupins: Their Unique Nutritional and Health-Promoting Attributes. Gluten-Free Ancient Grains.

[6] Yanez E., Ivanovic D., Owen D.F., Ballester D. (1983). Chemical and Nutritional Evaluation of Sweet Lupines. Ann. Nutr. Metab..

[7] Czubinski J., Wroblewska K., Czyzniejewski M., Górnaś P., Kachlicki P., Siger A. (2019). Bioaccessibility of Defatted Lupin Seed Phenolic Compounds in a Standardized Static *In Vitro* Digestion System. Food Res. Int..

[8] Kholif A.E., Olafadehan O.A. (2021). Essential Oils and Phytogenic Feed Additives in Ruminant Diet: Chemistry, Ruminal Microbiota and Fermentation, Feed Utilization and Productive Performance. Phytochem. Rev..

[9] Kholif A.E. (2023). A Review of Effect of Saponins on Ruminal Fermentation, Health and Performance of Ruminants. Vet. Sci..

[10] Um K.H., Shin J.S., Park B.K. (2023). Effect of Lupin Flake Supplementation on Rumen Fermentation and Meat Composition of Hanwoo Steers. S. Afr. J. Anim. Sci..

[11] Chaves A.V., He M.L., Yang W.Z., Hristov A.N., McAllister T.A., Benchaar C. (2008). Effects of Essential Oils on Proteolytic, Deaminative and Methanogenic Activities of Mixed Ruminal Bacteria. Can. J. Anim. Sci..

[12] Sallam S.M.A., Bueno I.C.S., Brigide P., Godoy P.B., Vitti D.M.S., Abdalla A.L., Papachristou T.G., Parissi Z.M., Ben Salem H., Morand-Fehr P. (2009). Production in Efficacy of Eucalyptus Oil on *In Vitro* Ruminal Fermentation and Methane Production. Options Méditerranéennes: Série A. Séminaires Méditerranéens.

[13] Kumar R., Kamra D.N., Agarwal N., Chaudhary L.C. (2009). Effect of Eucalyptus (*Eucalyptus globulus*) Oil on *In Vitro* Methanogenesis and Fermentation of Feed with Buffalo Rumen Liquor. Anim. Nutr. Feed Technol..

[14] Hynd P.I., Valentine S.C., Bartsch B.D. (1985). Rumen Protozoa Numbers in Dairy Cows Fed Barley or Lupins. Proc. Nutr. Soc..

[15] Patra A., Park T., Kim M., Yu Z. (2017). Rumen Methanogens and Mitigation of Methane Emission by Anti-Methanogenic Compounds and Substances. J. Anim. Sci. Biotechnol..

[16] Obeidat B.S., Kridli R.T., Ata M., Mahmoud K.Z., Bartlewski P.M. (2021). Nutrient Intake, in Vivo Digestibility, Growth Performance and Carcass Quality of Growing Lambs Fed Concentrate Diets Containing Sweet Lupin Grain (*Lupinus angustifolius*). Small Rumin. Res..

[17] Kholif A.E., Olafadehan O.A., Gouda G.A., Fahmy M., Morsy T.A., Ammar H., Hamdon H.A., Chahine M. (2024). Turmeric Rhizomes Reduced *In Vitro* Methane Production and Improved Gas Production and Nutrient Degradability. Anim. Biotechnol..

[18] Kholif A.E., Gouda G.A., Fahmy M., Morsy T.A., Abdelsattar M.M., Vargas-Bello-Pérez E. (2024). Fennel Seeds Dietary Inclusion as a Sustainable Approach to Reduce Methane Production and Improve Nutrient Utilization and Ruminal Fermentation. Anim. Sci. J..

[19] Kholif A.E., Rahman M.A., Abo El-Nor S.A.H., Morsy T.A., Gouda G.A., Fahmy M., Chahine M. (2024). Efficacy of *Salvia officinalis* Shrub as a Sustainable Feed Additive for Reducing Ruminal Methane Production and Enhancing Fermentation in Ruminants. Animals.

[20] Qin D.-M., Wang X.-B., Zou N., Han C., Xu J. (2019). Gas Chromatography-Mass Spectrometry (GC-MS) Analysis of the Volatile Oil of *Cichorium Glandulosum Boiss et Huet* and Its Effects on Carbon Tetrachloride-Induced Liver Fibrosis in Rats. Med. Sci. Monit..

[21] Goering H.K., Van Soest P.J. (1975). Forage Fiber Analyses.

[22] Fortina R., Glorio Patrucco S., Barbera S., Tassone S. (2022). Rumen Fluid from Slaughtered Animals: A Standardized Procedure for Sampling, Storage and Use in Digestibility Trials. Methods Protoc..

[23] AOAC (1997). Official Methods of Analysis of AOAC International.

[24] Van Soest P.J., Robertson J.B., Lewis B.A. (1991). Methods for Dietary Fiber, Neutral Detergent Fiber, and Nonstarch Polysaccharides in Relation to Animal Nutrition. J. Dairy Sci..

[25] France J., Dijkstra J., Dhanoa M.S., Lopez S., Bannink A. (2000). Estimating the Extent of Degradation of Ruminant Feeds from a Description of Their Gas Production Profiles Observed *In Vitro*: Derivation of Models and Other Mathematical Considerations. Br. J. Nutr..

[26] Blümmel M., Steingaβ H., Becker K. (1997). The Relationship between *In Vitro* Gas Production, *In Vitro* Microbial Biomass Yield and 15 N Incorporation and Its Implications for the Prediction of Voluntary Feed Intake of Roughages. Br. J. Nutr..

[27] Menke K.H., Raab L., Salewski A., Steingass H., Fritz D., Schneider W. (1979). The Estimation of the Digestibility and Metabolizable Energy Content of Ruminant Feedingstuffs from the Gas Production When They Are Incubated with Rumen Liquor *In Vitro*. J. Agric. Sci..

[28] Tadele Y. (2015). White Lupin (*Lupinus albus*) Grain, a Potential Source of Protein for Ruminants: A Review. Res. J. Agric. Environ. Manag..

[29] Maia M.R.G., Monteiro A., Valente I.M., Sousa C., Miranda C., Castro C., Cortez P.P., Cabrita A.R.J., Trindade H., Fonseca A.J.M. (2023). Upcycling Post-Harvest Biomass Residues from Native European Lupinus Species: From Straws and Pod Shells Production to Nutritive Value and Alkaloids Content for Ruminant Animals. Front. Nutr..

[30] Ando S., Nishida T., Ishida M., Hosoda K., Bayaru E. (2003). Effect of Peppermint Feeding on the Digestibility, Ruminal Fermentation and Protozoa. Livest. Prod. Sci..

[31] Dixon R.M., Hosking B.J. (1992). Nutritional Value of Grain Legumes for Ruminants. Nutr. Res. Rev..

[32] Zdunczyk Z., Mikulski D., Jankowski J., Przybylska-Gornowicz B., Juskiewicz J. (2019). Gastrointestinal Response of Laying Hens to Graded Dietary Inclusion Levels of Yellow Lupine Seeds. Anim. Feed. Sci. Technol..

[33] Gresta F., Oteri M., Scordia D., Costale A., Armone R., Meineri G., Chiofalo B. (2023). White Lupin (*Lupinus albus* L.), an Alternative Legume for Animal Feeding in the Mediterranean Area. Agriculture.

[34] Bryszak M., Szumacher-Strabel M., Huang H., Pawlak P., Lechniak D., Kołodziejski P., Yanza Y.R., Patra A.K., Váradyová Z., Cieslak A. (2020). *Lupinus angustifolius* Seed Meal Supplemented to Dairy Cow Diet Improves Fatty Acid Composition in Milk and Mitigates Methane Production. Anim. Feed. Sci. Technol..

[35] Patra A.K. (2016). Recent Advances in Measurement and Dietary Mitigation of Enteric Methane Emissions in Ruminants. Front. Vet. Sci..

[36] Hristov A.N., Callaway T.R., Lee C., Dowd S.E. (2012). Rumen Bacterial, Archaeal, and Fungal Diversity of Dairy Cows in Response to Ingestion of Lauric or Myristic Acid. J. Anim. Sci..

[37] Abubakr A.R., Alimon A.R., Yaakub H., Abdullah N., Ivan M. (2013). Digestibility, Rumen Protozoa, and Ruminal Fermentation in Goats Receiving Dietary Palm Oil By-Products. J. Saudi Soc. Agric. Sci..

[38] Galagan J.E., Nusbaum C., Roy A., Endrizzi M.G., Macdonald P., FitzHugh W., Calvo S., Engels R., Smirnov S., Atnoor D. (2002). The Genome of M. Acetivorans Reveals Extensive Metabolic and Physiological Diversity. Genome Res..

[39] Song H., Clarke W.P., Blackall L.L. (2005). Concurrent Microscopic Observations and Activity Measurements of Cellulose Hydrolyzing and Methanogenic Populations during the Batch Anaerobic Digestion of Crystalline Cellulose. Biotechnol. Bioeng..

[40] Kholif A.E., Hassan A.A., El Ashry G.M., Bakr M.H., El-Zaiat H.M., Olafadehan O.A., Matloup O.H., Sallam S.M.A. (2021). Phytogenic Feed Additives Mixture Enhances the Lactational Performance, Feed Utilization and Ruminal Fermentation of Friesian Cows. Anim. Biotechnol..

[41] Elghandour M.M.Y., Kholif A.E., Salem A.Z.M., Montes de Oca R., Barbabosa A., Mariezcurrena M., Olafadehan O.A. (2016). Addressing Sustainable Ruminal Methane and Carbon Dioxide Emissions of Soybean Hulls by Organic Acid Salts. J. Clean. Prod..

[42] Garcia-Santos S., Almeida M., Closson M., Guedes C.M., Barros A., Ferreira L.M., Trindade H., Pinheiro V. (2021). Effect of Total Replacement of the Soya Bean Meal by Lupine Seeds (*L. Albus* and *L. Luteus*) on Performance and Digestion Characteristics of Growing Rabbits. Anim. Feed. Sci. Technol..

[43] Wang K., Xiong B., Zhao X. (2023). Could Propionate Formation Be Used to Reduce Enteric Methane Emission in Ruminants?. Sci. Total Environ..

[44] Park S.J., Beak S.H., Jung D.J.S., Kim S.Y., Jeong I.H., Piao M.Y., Kang H.J., Fassah D.M., Na S.W., Yoo S.P. (2018). Genetic, Management, and Nutritional Factors Affecting Intramuscular Fat Deposition in Beef Cattle—A Review. Asian-Australas. J. Anim. Sci..

[45] Akhtar M., Chen Y., Ma Z., Zhang X., Shi D., Khan J.A., Liu H. (2022). Gut Microbiota-Derived Short Chain Fatty Acids Are Potential Mediators in Gut Inflammation. Anim. Nutr..

[46] Maheri-Sis N., Chamani M., Sadeghi A.-A., Mirza-Aghazadeh A., Aghajanzadeh-Golshani A. (2008). Nutritional Evaluation of Kabuli and Desi Type Chickpeas (*Cicer arietinum* L.) for Ruminants Using *In Vitro* Gas Production Technique. Afr. J. Biotechnol..

[47] Azar M.S., Doust-Nobar R.S., Sis N.M., Shahryar H.A., Asadi Y. (2012). Effects of *Zataria multiflora* Extract as Rumen Modifiers Using *In Vitro* Gas Production Technique. Curr. Res. J. Biol. Sci..

[48] Fellner V. (2004). Rumen Microbes and Nutrient Management.

[49] Almeida M., Garcia-Santos S., Nunes A., Rito S., Azevedo J., Guedes C., Silva S., Ferreira L. (2021). Introducing Mediterranean Lupins in Lambs’ Diets: Effects on Growth and Digestibility. Animals.

[50] Gomaa A.S., Kholif A.E., Kholif A.M., Salama R., El-Alamy H.A., Olafadehan O.A. (2018). Sunflower Oil and *Nannochloropsis oculata* Microalgae as Sources of Unsaturated Fatty Acids for Mitigation of Methane Production and Enhancing Diets’ Nutritive Value. J. Agric. Food Chem..

[51] Guo T., Guo T., Cao Y., Guo L., Li F., Li F., Yang G. (2021). Changes in the Fermentation and Bacterial Community by Artificial Saliva pH in RUSITEC System. Front. Nutr..

[52] Dijkstra J., Ellis J.L., Kebreab E., Strathe A.B., López S., France J., Bannink A. (2012). Ruminal pH Regulation and Nutritional Consequences of Low pH. Anim. Feed. Sci. Technol..

[53] Laporte-Uribe J.A. (2016). The Role of Dissolved Carbon Dioxide in Both the Decline in Rumen pH and Nutritional Diseases in Ruminants. Anim. Feed. Sci. Technol..

[54] Kamra D.N. (2005). Rumen Microbial Ecosystem. Curr. Sci..

[55] Ososanya T.O., Odubola O.T., Shuaib-Rahim A. (2013). Intake, Nutrient Digestibility and Rumen Ecology of West African Dwarf Sheep Fed Palm Kernel Oil and Wheat Offal Supplemented Diets. Int. J. Agrisci..

[56] Salem A.Z., Kholif A.E., Elghandour M.M.Y., Hernandez S.R., Domínguez-Vara I.A., Mellado M. (2014). Effect of Increasing Levels of Seven Tree Species Extracts Added to a High Concentrate Diet on *In Vitro* Rumen Gas Output. Anim. Sci. J..

[57] Elghandour M.M.Y., Kholif A.E., Salem A.Z.M., Olafadehan O.A., Kholif A.M. (2016). Sustainable Anaerobic Rumen Methane and Carbon Dioxide Productions from Prickly Pear Cactus Flour by Organic Acid Salts Addition. J. Clean. Prod..

[58] Olafadehan O.A., Adebayo O.F. (2016). Nutritional Evaluation of Ammoniated Ensiled Threshed Sorghum Top as a Feed for Goats. Trop. Anim. Health Prod..

[59] Um K.H., Shin J.S., Son G.H., Park B.K. (2024). Effect of Lupin Supplementation on the Growth, Carcass, and Meat Characteristics of Late-Fattening Hanwoo Steers. Animals.

[60] Aguilar-Hernández J.A., Urías-Estrada J.D., López-Soto M.A., Barreras A., Plascencia A., Montaño M., González-Vizcarra V.M., Estrada-Angulo A., Castro-Pérez B.I., Barajas R. (2016). Evaluation of Isoquinoline Alkaloid Supplementation Levels on Ruminal Fermentation, Characteristics of Digestion, and Microbial Protein Synthesis in Steers Fed a High-Energy Diet. J. Anim. Sci..

[61] Thirumalesh T., Krishnamoorthy U. (2013). Rumen Microbial Biomass Synthesis and Its Importance in Ruminant Production. Int. J. Livest. Res..

[62] Rodríguez R., Sosa A., Rodríguez Y. (2007). Microbial Protein Synthesis in Rumen and Its Importance to Ruminants. Cuba. J. Agric. Sci..

